# Reproductive physiology of the heat-stressed dairy cow: implications for fertility and assisted reproduction

**DOI:** 10.21451/1984-3143-AR2019-0053

**Published:** 2019-10-23

**Authors:** Peter J. Hansen

**Affiliations:** Department of Animal Sciences, D. H. Barron Reproductive and Perinatal Biology Research Program and Genetics Institute, University of Florida, Gainesville, Florida, USA.

**Keywords:** heat stress, lactating cow, reproduction, fertility, embryo, oocyte

## Abstract

Heat stress causes a large decline in pregnancy success per insemination during warm times of the year. Improvements in fertility are possible by exploiting knowledge about how heat stress affects the reproductive process. The oocyte can be damaged by heat stress at the earliest stages of folliculogenesis and remains sensitive to heat stress in the peri-ovulatory period. Changes in oocyte quality due to heat stress are the result of altered patterns of folliculogenesis and, possibly, direct effects of elevated body temperature on the oocyte. While adverse effects of elevated temperature on the oocyte have been observed *in vitro*, local cooling of the ovary and protective effects of follicular fluid may limit these actions *in vivo*. Heat stress can also compromise fertilization rate. The first seven days of embryonic development are very susceptible to disruption by heat stress. During these seven days, the embryo undergoes a rapid change in sensitivity to heat stress from being very sensitive (2- to 4-cell stage) to largely resistant (by the morulae stage). Direct actions of elevated temperature on the embryo are likely to be an important mechanism for reduction in embryonic survival caused by heat stress. An effective way to avoid effects of heat stress on the oocyte, fertilization, and early embryo is to bypass the effects through embryo transfer because embryos are typically transferred into females after acquisition of thermal resistance. There may be some opportunity to mitigate effects of heat stress by feeding antioxidants or regulating the endocrine environment of the cow but neither approach has been reduced to practice. The best long-term solution to the problem of heat stress may be to increase genetic resistance of cows to heat stress. Thermotolerance genes exist within dairy breeds and additional genes can be introgressed from other breeds by traditional means or gene editing.

## Introduction

**Figure 1:**
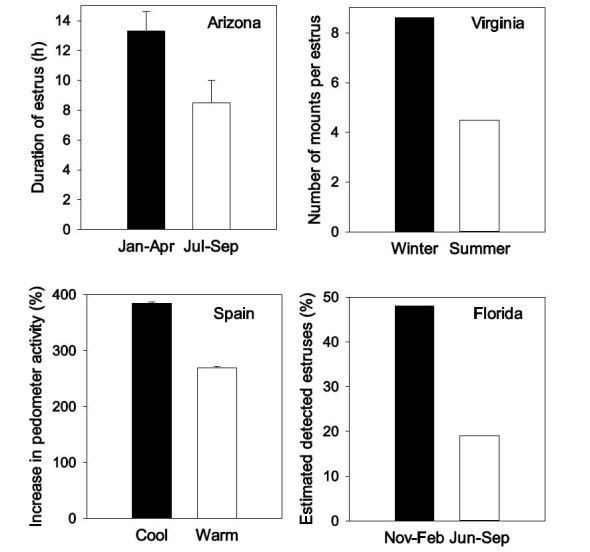
Seasonal variation in characteristics of estrus in lactating cows. Shown are data on duration of estrus in Arizona (Wolff and Monty, 1974), number of mounts per estrus in Virginia (Nebel *et al*., 1997), the increase in pedometer activity at estrus in Spain (López-Gatius *et al*., 2005a) and estimated percent of estrus periods detected by farm personnel in Florida (Thatcher *et al*., 1986). The figure is reproduced from Hansen (2017) with permission of the American Dairy Science Association.

**Figure 2:**
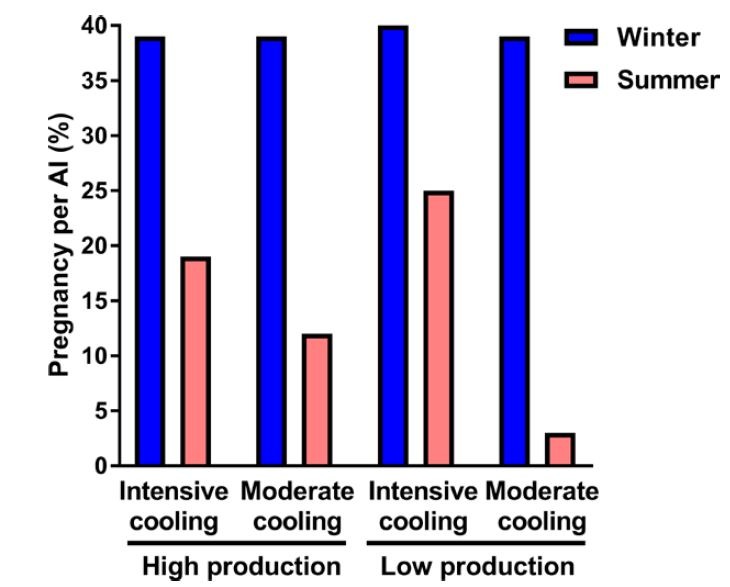
Pregnancies per artificial insemination of herds surveyed in Israel (Flamenbaum and Galon, 2010). Herds were classified based on the overall level of milk production (high *vs* low) and on the degree of cooling that cows receive (intensive *vs* moderate).

The overall reproductive function of a herd of dairy cows is often estimated by calculating pregnancy rate, i.e., the product of estrus detection rate (how many cows in estrus are detected in estrus by farm personnel) and conception rate (a misnomer but a measure of how many cows that are inseminated are diagnosed as pregnant). A pregnancy rate of 100% would mean that every cow eligible to be pregnant in a 21-day period becomes pregnant in that time. By this measure, the reproductive function of the heat-stressed dairy cow can be very low indeed. Data in Figure 1 illustrate how heat stress can affect characteristics of estrous activity; only 19% of estrus periods were detected by farm workers in the summer in one study in Florida (Thatcher *et al*., 1986). Fertility after artificial insemination (AI) can also be low during heat stress. In a survey of dairy herds in Israel, less than 20% of inseminations resulted in pregnancies in the summer and pregnancy per AI (P/AI) in the worst herds (those with milk low production and a moderate amount of cooling) was only 3% (Fig. 2; Flamenbaum and Galon, 2010). In another study, P/AI at day 32 after insemination for lactating cows in Minais Gerais, Brazil was 17% when cows experienced two of more occurrences of a morning rectal temperature greater than 39.1°C at days -3, -2, 0 and 7 relative to timed AI *vs* 25% for cows with one occurrence and 37% for cows with no occurrence (Pereira *et al*., 2013).

Fortunately, the situation is not always so bleak. For example, P/AI at day 36 after insemination for multiparous cows in Oklahoma and Kansas during hot weather was 25-27% (Voelz *et al*., 2016). In a study in Florida, P/AI for multiparous cows in the summer on a free-stall dairy in Florida with fans and sprinkler was 32% (Zolini *et al*., 2019). Higher pregnancy rates during heat stress can be ascribed to superior cooling systems and implementation of timed AI programs. Not only do protocols for timed AI eliminate the need for estrus detection but, for some protocols, they can increase cow fertility (Carvalho *et al*., 2018).

Further improvements in fertility during the summer are possible by exploiting knowledge about how heat stress affects the reproductive process. Here, the impact of heat stress on follicular development, oocyte quality, fertilization, and embryonic development will be briefly outlined and the consequences of those changes for strategies to improve fertility will be discussed. Since AI using frozen semen can bypass effects on the bull, and timed AI bypasses effects on estrus behavior, the focus will be on biological processes important for establishing a high level of fertility. Keep in mind that there are effects of heat stress after establishment of pregnancy, most notably in late gestation when heat stress can affect milk yield of the cow after calving and the epigenetic program, growth and milk yield of the resulting calf (Dahl *et al*., 2017; Skiebiel *et al*., 2018). However, this important aspect of actions of heat stress on the pregnant cow is beyond the scope of the current review.

## Reproductive Events Susceptible to Heat Stress

There have been attempts to reduce the impact of heat stress by cooling lactating cows for a limited period of time around ovulation, fertilization and early development but the improvement in fertility has been slight (Stott and Wiersma, 1976; Her *et al*., 1988; Ealy *et al*., 1994). This is because there is a broad window of time in which the reproductive process of the cow can be disrupted by heat stress. An experiment with Gir cattle indicates that heat stress can compromise development of the oocyte at the earliest stages of folliculogenesis. In particular, competence of oocytes to develop to the blastocyst stage after *in vitro* fertilization was reduced by heat stress occurring as early as 105 to 133 days before oocyte retrieval (Torres-Júnior *et al*., 2008). In an experiment with lactating Holsteins, secretion of androstenedione from cultured thecal cells and estradiol from cultured granulosa cells from medi,um-sized follicles was reduced by exposure of donor cows to heat stress 20 to 26 days earlier (Roth *et al*., 2001a). Additional evidence that heat stress affects the oocyte early in folliculogenesis are observations that oocyte competence for supporting embryonic development after *in vitro* activation was restored only gradually after the end of summer (see review by Hansen, 2013a; Roth, 2017). Moreover, treatments to increase follicular turnover, including multiple follicular aspirations (Roth *et al*., 2001b), follicle stimulating hormone (Roth *et al*., 2002) or somatotropin (Roth *et al*., 2002), hastened restoration of oocyte competence.

The oocyte remains sensitive to heat stress in the peri-ovulatory period. Treatment of superovulated cows with heat stress for 10 hours beginning at the onset of estrus reduced the percent of embryos recovered at day 7 after estrus that were classified as having normal morphology and increased the percent of embryos that were retarded with development (Putney *et al*., 1989a).

Heat stress can compromise fertilization rate (Sartori *et al*., 2002; Hackbart *et al*., 2010). For example, fertilization rate in lactating cows bred by AI was lowered from 88% in winter to 55% in summer (Sartori *et al*., 2002). Low fertilization rates could reflect damage to the oocyte, sperm deposited in the uterus, or disruption of the fertilization process itself. Effects on the sperm or fertilization process may be a more important cause than effects on the oocyte. Competence of the oocyte to be fertilized *in vitro* was not reduced in the summer although there was a reduction in development of cleaved embryos to the blastocyst stage (Ferreira *et al*., 2011). Further, indirect evidence for an effect of heat stress on the sperm is the observation of Girolando cows that insemination in the morning was associated with a slight but significant increase in P/AI (Rocha de Zouza *et al*., 2016).

The first 7 days of embryonic development are very susceptible to disruption by heat stress. Experimental treatment of superovulated cows with heat stress during this time reduced the development of embryos at day 7 (Putney *et al*, 1988a). Moreover, among single-ovulating lactating cows, there was a large reduction in the percent of recovered embryos classified as viable during periods of heat stress as compared to periods of no heat stress (Sartori *et al*., 2002). During these 7 days, the embryo undergoes a rapid change in sensitivity to heat stress. Exposure of superovulated cows to heat stress on day 1 after estrus reduced the percent of embryos that were blastocysts at day 8 (Ealy *et al*., 1993). However, heat stress at day 3, 5 or 7 had no effect on embryonic development. Thus, the resistance of the embryo to heat stress increases greatly in just a few days of development.

There is little known about sensitivity of the bovine embryo to heat stress after day 7. There is one report in beef cattle that heat stress from day 8 to 16 can reduce conceptus weight at day 16 (Biggers *et al*., 1987). However, the effectiveness of embryo transfer as a tool for improving fertility during heat stress (see discussion further in this paper) is indicative that embryo survival is not dependent to any large extent on the occurrence of maternal heat stress after day 7.

A proportion of cows initially diagnosed as pregnant around day 28-60 of pregnancy subsequently lose the pregnancy. There is some evidence that the frequency of this loss can be increased by heat stress (García-Ispierto *et al*., 2006; Santolaria *et al*., 2010; El-Tarabany and El-Tarabany, 2015). For example, pregnancy loss between days 34 to 45 of gestation and day 90 of gestation were 2% for cows in the cool season *vs* 12% for cows in the warm season (García-Ispierto *et al*., 2006). Attempts have been made to understand the crucial period in the reproductive process during which heat stress acts to increase late embryonic and fetal mortality by relating indices of heat stress at specific phases in the reproductive process to pregnancy loss. Such an approach is difficult to decipher because environmental conditions at one period are often highly correlated with environmental conditions at another period.

## Physiological Causes of Effects of Heat Stress on the Oocyte and Embryo

Effects of heat stress are related to the inability of the affected cow to maintain its body temperature within the regulated range. As mentioned, P/AI is related to rectal temperature (Pereira *et al*., 2013). It has been estimated that fertility begins to decline when uterine temperature rises about 0.5°C above normal (Gwazdauskas *et al*., 1973). One reason why lactating cows are more susceptible to the negative effects of heat stress on fertility than heifers (Badinga *et al*., 1985) is because the metabolic heat production associated with lactation makes it more difficult for cows to regulate body temperature during heat stress than non-lactating heifers (Sartori *et al*., 2002). Effects of heat stress on the ovary, oviduct, uterus, and embryo could result from either physiological changes caused by heat stress or by the direct effects of elevated temperature on cells involved in reproduction.

Alterations in oocyte quality due to heat stress probably involve deviations in patterns of folliculogenesis. Follicular dominance is reduced in cows exposed to heat stress so that there is an increase in number of large follicles on the ovary, prolonged period of dominance of the ovulatory follicle, increased circulating concentrations of follicle stimulating hormone (FSH) and reduced concentrations of estradiol-17β and inhibin (Wolfenson *et al*., 1995; Roth *et al*., 2000; Trout *et al*., 1998; Wilson *et al*., 1998). Heat stress can also dampen the preovulatory surge of luteinizing hormone and estradiol-17β (Gwazdauskas *et al*., 1981; Gilad *et al*., 1993; Armengol-Gelonch *et al*., 2017). Indeed, heat stress can increase the proportion of cows that fail to ovulate after administration of GnRH. Ovulation failure was 12% during the warm period *vs* 3% during the cool period (López-Gatius *et al*., 2005b). Use of more active analogs of gonadotropin releasing hormone (GnRH) can reduce the incidence of ovulation failure (García-Ispierto *et al*., 2019).

There are direct effects of elevated temperature (i.e., heat shock) on the competence of the oocyte undergoing maturation to develop into a blastocyst following fertilization or artificial activation (see Roth, 2017 for review). Possible local cooling of the ovary and protective effects of follicular fluid may limit these actions *in vivo*. Work by López-Gatius and Hunter (2017, 2019a,b) has revealed that the ovary experiences a cooler temperature than that measured in the rectum or on the surface of the uterus. Additionally, culture of maturing oocytes in a medium containing follicular fluid or follicular fluid exosomes reduced the negative effect of elevated temperature on oocyte competence for cleavage and blastocyst development after fertilization (Rodrigues *et al*., 2019). Direct effects of elevated temperature on the follicle may be important in some circumstances, however. Cows in which follicular temperature was lower than rectal temperature were more likely to ovulate and achieve pregnancy than cows in which the gradient between follicular and rectal temperature was low (López-Gatius and Hunter, 2019ab).

Direct actions of elevated temperature on the embryo are likely to be an important mechanism for reduction in embryonic survival caused by heat stress after ovulation. Indeed, the changes in embryonic resistance to maternal heat stress observed *in vivo* (Ealy *et al*., 1993) are also seen with effects of heat shock on cultured embryos. Exposure of the zygote and 2-cell embryo causes a large reduction in percent of embryos developing to the blastocyst stage (Edwards and Hansen, 1997; Sakatani *et al*., 2012; Ortega *et al*., 2016). Embryos at the 4- and 8-cell stage are also susceptible to heat shock but the magnitude of the deleterious effect is reduced as compared to that for the 2-cell embryo (Edwards and Hansen, 1997). Physiologically-relevant heat shock has little effect on development of morula-stage embryos (Edwards and Hansen, 1997; Eberhardt *et al*., 2009; Sakatani *et al*., 2012). Mechanisms responsible for acquisition of thermotolerance are not known but probably involve activation of the embryonic genome at the 8-cell stage (Graf *et al*., 2014) so that the full range of cellular adaptations to heat shock can be employed.

It is also possible that changes in circulating concentrations of steroid hormones induced by heat stress could alter the oviductal or uterine environment and thereby affect embryonic development. As stated previously, heat stress can reduce plasma concentrations of estradiol-17β (Gwazdauskas *et al*., 1981; Wolfenson *et al*., 1995; Wilson *et al*., 1998). Short-term exposure to heat stress either had no effect on plasma concentrations of progesterone (Roth *et al*., 2000) or caused an increase (Trout *et al*., 1998; Wilson *et al*., 1998). Long-term exposure to heat stress may lead to reduced progesterone concentrations, however, because luteal concentrations of the hormone during the luteal phase have been reported to be lower in summer than winter (Howell *et al*., 1994). Additionally, cooling cows during the summer increased circulating concentrations of progesterone (Wolfenson *et al*., 1988). Some effects of heat stress on peripheral blood concentrations of hormones could be the result of changes in water balance during heat stress and reduced hematocrit (Richards, 1985; Lamp *et al*., 2015).

## Embryo Transfer: The Most Effective Mechanism for Maximizing Fertility During Heat Stress

One way to avoid consequences of heat stress on the oocyte, fertilization, and early embryo is to bypass its effects through implementation of an embryo transfer program. Embryos are typically transferred into females at day 7 after estrus. By that time, embryos have gained resistance to effects of heat stress. Embryo transfer can be coupled with ovulation synchronization programs to allow timed embryo transfer and avoid the need for estrus detection.

**Figure 3:**
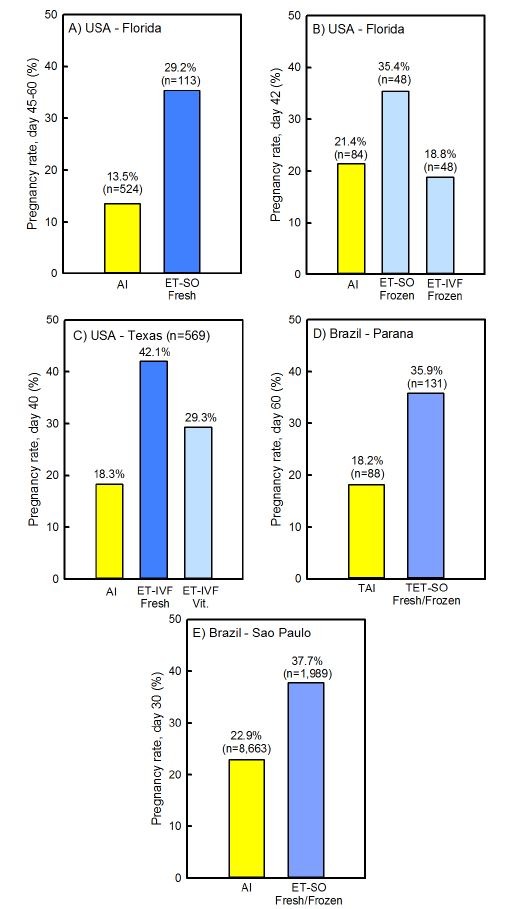
Comparisons of pregnancy success for artificial insemination *vs* embryo transfer in the summer. Data are from Putney *et al*. (1989b) (A), Drost *et al*. (1999) (B), Stewart *et al*. (2011) (C), Vasconcelos *et al*. (2011) (D) and Baruselli *et al*. (2011) (E). Abbreviations are as follows: AI, artificial insemination, ET, embryo transfer; IVF, *in vitro* fertilized; SO, superovulation; TAI, timed AI; TET, timed embryo transfer; Vit., vitrified. The figure is modified from a technical bulletin by Vetoquinol and is reproduced with permission.

One way to demonstrate the effectiveness of embryo transfer for improving fertility during heat stress is to compare pregnancy outcomes for embryo transfer as compared to AI. As summarized in Figure 3, pregnancy rates during heat stress have been consistently higher for cows receiving an embryo than for cows submitted to AI. The only exception is when embryos were produced *in vitro* and cryopreserved before transfer. Thus, there was either no improvement in fertility as compared to AI when vitrified embryos produced *in vitro* were transferred (Drost *et al*., 1999; Fig. 3B) or the improvement was less than if fresh embryos were transferred (Stewart *et al*., 2011; Fig. 3C). These results are the consequence of poor cryopreservation of *in vitro* produced embryos (Hansen and Block, 2004).

**Figure 4:**
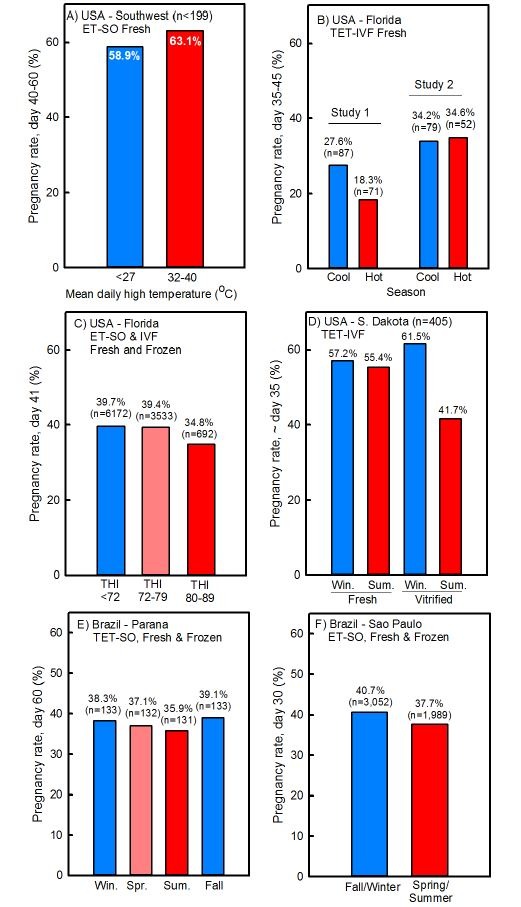
Comparisons of percent cows pregnant following embryo transfer in cool or hot weather. Data are from Putney *et al*. (1988b) (A), Block *et al*. (2007) and Loureiro *et al*. (2009) (B), Ferraz *et al*. (2016) (C), Chebel *et al*. (2008) (D), Vasconcelos *et al*. (2011) (E) and Baruselli *et al*. (2011) (F). Abbreviations are as follows: ET, embryo transfer; IVF, *in vitro* fertilized; S. Dakota, South Dakota; Spr., spring; SO, superovulation; Sum., summer; TET, timed embryo transfer; THI, temperature-humidity index; Win., winter. The figure is modified from a technical bulletin by Vetoquinol and is reproduced with permission.

Another way to demonstrate how embryo transfer reduces the impact of heat stress is to examine seasonal variation in pregnancy success after embryo transfer. Of eight studies in which seasonal variation in pregnancy rates were evaluated, there were only two cases where there was a large difference in pregnancy rate between hot and cool conditions including an experiment in Florida with fresh embryos produced *in vitro* (Block *et al*., 2007; Fig. 4B) and an experiment in South Dakota with vitrified embryos produced *in vitro* (Chebel *et al*., 2008; Fig. 4C). There was no difference between seasons for embryos produced by superovulation in the southwest United States (Putney *et al*., 1988b; Fig. 4A) or for fresh embryos produced *in vitro* in Florida (Loureiro *et al*., 2009; Fig. 4B) or South Dakota (Chebel *et al*., 2008; Fig. 4C). In the largest trials, there was a slight reduction in pregnancy per embryo transfer in the hottest months (Ferraz *et al*., 2016; Vasconcelos *et al*., 2011; Baruselli *et al*., 2011) but the difference in pregnancy outcomes between the coolest and warmest times were only 3 to 4% (Fig. 444F). Seasonal variation of that magnitude is much less than what would be the case for AI.

## Antioxidants

Exposure to elevated temperature can increase production of reaction oxygen species in oocytes (Nabenishi *et al*., 2012; Cavallari de Castro *et al*., 2019) and embryos (Sakatani *et al*., 2004; Sakatani *et al*., 2008; Ortega *et al*., 2016). *In vitro*, effects of heat shock on oocyte maturation can be reduced by provision of antioxidants (Lawrence *et al*., 2004; Nabenishi *et al*., 2012; Ispada *et al*., 2018; Cavallari de Castro *et al*., 2019). In the embryo, however, thermoprotective benefits of antioxidants *in vitro* have been inconsistent. There was no protective effect of dithiothreitol, glutathione, melatonin, taurine, or vitamin E (Ealy *et al*., 1995; Paula-Lopes *et al*., 2003a; de Castro *et al*., 2008; Ortega *et al*., 2016) but β-mercaptoethanol was protective (Sakatani *et al*., 2008). One interpretation of these data is that reactive oxygen species are a more important mediator of the embryotoxic actions of heat shock for the oocyte than the embryo.

Efforts to improve fertility of lactating cows exposed to heat stress by delivering antioxidants have generally not yielded positive effects (see review by Hansen, 2013b and Roth, 2017). There are two reports of beneficial effects of antioxidant administration, however. In the first, Aréchiga et al. (1998) found that a higher proportion of cows fed supplemental β-carotene from about day 15 after calving were pregnant at 90 d postpartum than control cows when the experiment was performed during the summer but not when the experiment was performed during the winter. Feeding supplemental β-carotene did not increase pregnancy per AI at first service so the effect on the proportion pregnant at 90 d reflects either alterations in fertility after first service or estrus detection. In the second report, administration of long-acting melatonin implants beginning at 220 d of gestation to cows during the summer shortened the interval to conception in the subsequent postpartum period and decreased the incidence of cows experiencing > 3 breedings per conception (García-Ispierto *et al*., 2013). The peak concentration of melatonin in the blood of cows receiving implants was low (i..e, 260-300 pM) and it might be that melatonin was acting as a hormone rather than as an antioxidant. Further studies are needed with both β-carotene and melatonin to evaluate efficacy of their administration for improving fertility during heat stress.

## Hormonal Treatments

Much work continues on optimization of timed AI protocols in general and under the specific conditions of heat stress. Few studies have been performed to compare whether a specific improvement in a timed AI protocol works better for heat-stressed cows than cows not subjected to heat stress. One exception is for induction of ovulation. As already discussed, ovulation failure is more frequent during periods of heat stress and administration of more active analogs of GnRH can reduce the incidence of ovulation failure (García-Ispierto *et al*., 2019). In another study, Shabankareh *et al*. (2010) evaluated the summer-winter differences in P/AI at first service for cows bred at spontaneous estrus or following timed AI using either GnRH (Ovsynch) or estradiol cypionate (Heatsynch) to induce ovulation. There was no difference in P/AI (32, 30 and 30% for OvSynch, Heatsynch and spontaneous estrus) in the summer while P/AI in the winter was highest for spontaneous estrus (51%), intermediate in Ovsynch (40%) and lowest for Heatsynch (35%).

Several experiments have been conducted to evaluate effects of increasing circulating progesterone concentrations on fertility of heat-stressed cows. Results have been inconsistent and often dependent on the subset of cows treated. Administration of progesterone using a CIDR device from day 5 to 18 after insemination did not cause an overall increase in P/AI but there were positive effects of the treatment in cows with low body condition or postpartum uterine disorders (Friedman *et al*., 2012). In the study of Shabankareh *et al*. (2010), treatment with human chorionic gonadotropin (hCG) on day 5 after insemination increased P/AI in both summer (24 *vs* 38% for saline and hCG) and winter (35 *vs* 47%). Treatment with hCG at day 5 also increased pregnancy rate in cows during summer but the effect was seen only for primiparous cows (Zolini *et al*., 2019). Treatment with GnRH at AI or at both AI and day 12 of the estrous cycle increased P/AI in an experiment by López-Gatius *et al*. (2005c). In another research trial, there was no beneficial effect of treatment at day 0 on P/AI whereas treatment with GnRH at either day 5 or both day 0 and 5 increased P/AI but only for cows in third or greater lactation (Mendonça *et al*., 2017).

The idea that ovarian follicles can be compromised by heat stress at early stages of folliculogenesis has lead to the idea that fertility can be improved in the autumn by hastening the removal of damaged follicles from the ovary. Improved oocyte competence in the autumn, as measured by *in vitro* development to the blastocyst stage, has been achieved using several treatments to increase follicular turnover, including multiple follicular aspirations (Roth *et al*., 2001b) or treatment with FSH (Roth *et al*., 2002) or somatotropin (Roth *et al*., 2002). In addition, generation of three consecutive 9-day follicular waves by treatment with GnRH and prostaglandin F_2α_ has been reported to have some positive effects on fertility of lactating cows in the summer and autumn (Friedman *et al*., 2011). Treatment effects were seen for primiparous cows (37% *vs* 53% for control and treated cows) but not for multiparous cows (27 *vs* 29%).

## Genetic Selection

Heritability estimates in Holsteins for body temperature during heat stress is 0.17 (Dikmen *et al*., 2012) and for the decline in milk yield during heat stress is 0.19 (Nguyen *et al*., 2016). Thus, it should be possible to reduce the impact of heat stress on reproduction by selecting genetically for thermoregulation. Data from Australia indicate that cows that are more thermotolerant genetically also have higher breeding values for fertility (Nguyen *et al*., 2016). Unfortunately, they also have a lower genetic ability for milk yield so genetic strategies must be developed to allow selection for genes that confer superior thermotolerance without compromising milk yield.

One option is to introgress genes from thermotolerant breeds into dairy breeds using crossbreeding or gene editing. The prolactin receptor gene is one gene that has been mutated in a manner that leads to a slick hair phenotype characterized by a sleek, short hair coat and increased capacity for regulating body temperature (Dikmen *et al*., 2014). Arising in criollo breeds of cattle, several mutations in the gene exist that result in a truncated version of the protein to be synthesized (Porto-Neto *et al*., 2018). The gene has been introduced into Holsteins and is associated with reduced milk yield depression in the summer (Dikmen *et al*., 2014). Data from Puerto Rico indicate that slick-haired Holsteins are more fertile than Holsteins without the mutation (Ortiz-Colón *et al*., 2018).

There are also genetic effects on cellular resistance to elevated temperature. Embryos from *Bos indicus* breeds or the Romosinuano, a criollo breed, are more resistant to deleterious effects of heat shock on development of cultured embryos (Paula-Lopes *et al*., 2003b; Hernández-Cerón *et al*., 2004; Eberhardt *et al*., 2009; Silva *et al*., 2013). Fertility of cows in the summer was higher when inseminations were performed with Gyr semen than when Holstein semen was used (Pegorer *et al*., 2007). One gene that contains mutations that increases cellular resistance to heat shock is *HSPA1L*, as indicated by studies with lymphocytes (Basiricò *et al*., 2011) and embryos (Ortega *et al*., 2016).

## Final Note

The decision as to which strategies to implement to reduce effects of heat stress on fertility is not a simple one. Embryo transfer, for example, while effective at minimizing the summer decline in fertility, is also expensive and may not be economically-effective unless the cost is constrained. In addition, getting cows pregnant in the summer can have long-term negative consequences for the resultant calf. Pinedo and De Vries (2017) have demonstrated that cows conceived in summer were older at first calving, had lower odds of surviving for a second calving, longer intervals from calving to first breeding and conception, and lower milk yield than cows conceived in winter. Thus, in some cases, non-uniform or seasonal calving may be the most profitable strategy. Genetic strategies that increase thermotolerance of the cow population are also desirable because, among other reasons, effects are permanent for that animal and extend to its offspring.
